# Fulminant emphysematous hepatitis with pneumoperitoneum: A case report

**DOI:** 10.1016/j.radcr.2026.07.109

**Published:** 2026-08-25

**Authors:** Jonathan Samuel Perkins, Paymon Zomorodian, Vamsidhar Rachapalli

**Affiliations:** a Royal Liverpool Hospital, Liverpool University Hospitals Foundation NHS Trust, Liverpool L7 8YE, UK; b Whiston Hospital, Mersey & West Lancashire Teaching Hospitals NHS Trust, Prescot, L35 5DR, UK

**Keywords:** Emphysematous hepatitis, Liver abscess, Hepatic abscess, Liver, Computed tomography

## Abstract

Emphysematous hepatitis is a rare and fulminant gas-forming intra-abdominal infection with a high rate of mortality. Concurrent Type II Diabetes Mellitus is the most common association. It is diagnosed through CT examination with replacement of the liver parenchyma with gas without fluid-containing abscess formation. We describe the case of a gentleman in his sixties presenting with acute abdominal pain, fever and jaundice diagnosed with emphysematous hepatitis via CT with a rapidly progressive and terminal course. The imaging demonstrated large volume intrahepatic gas with pneumoperitoneum. The underlying pathogens isolated were *Enterococcus faecium* and *Clostridium perfringens*. This is believed to be the 26th case described in the literature with many similarities to previously published cases.

## Introduction

Emphysematous hepatitis is a rapidly progressive condition with high morbidity and mortality that was only first described in the literature in 2002 [1]. It is characterized by replacement of the liver parenchyma with gas without significant fluid component or other features of pyogenic hepatic abscess such as wall enhancement and mass effect [2].

This exceedingly rare entity has been most commonly associated with a history of Type II Diabetes Mellitus (T2DM) with previous abdominal surgery and concurrent malignancy also being noted in several cases [3].

Through a search of the literature, we identified a total of 25 cases of emphysematous hepatitis that had previously been described with successful treatment in only 10 of these patients [4]. Until recently, all patients required intervention above and beyond intravenous antibiotics including percutaneous drainage and surgical intervention. A recent case was successfully treated with intravenous antibiotics alone [4].

We describe the case of a male patient in his sixties who presented to the emergency department who was subsequently diagnosed with emphysematous hepatitis via computed tomography (CT) imaging.

## Case report

A man in his sixties presented to the emergency department of a busy district general hospital with a 24-hour history of right sided abdominal pain. By the evening of presentation, this had worsened with radiation of the pain to the epigastrium. The patient also reported fever. On presentation the patient was clinically found to be jaundiced.

The patient had a history of a hiatus hernia. Although previously treated for gout, this was subsequently investigated and diagnosed as osteoarthritis following no response to allopurinol. At the time of presentation, the patient was taking metformin for T2DM but had been recently reviewed at his Primary Care Centre for this and due to recent HbA1c results, had discussed a reduction in dose and potential cessation of metformin therapy. The patient declined this at the time with a plan to review again at a follow up appointment. There was no history of previous abdominal surgery or prior malignancy. Following a couple years history of right upper quadrant pain, an ultrasound examination had raised the possibility of gallstones.

Initial assessment of the patient in the emergency department identified marked physiological compromise. Observations demonstrated a temperature of 39.9°C, tachycardia with a rate of 130 beats per minute and tachypnoea with a rate of 40 breaths per minute. There was no hypotension or reduction in oxygen saturations at this time. Clinical examination revealed tenderness to palpation of the right upper quadrant and the epigastrium. He was noted to be drowsy and lethargic with jaundiced sclera. Initial working diagnosis was that of acute cholecystitis with common bile duct obstruction, most likely due to gallstones.

Blood samples were obtained but all biochemical parameters could not be analyzed due to hemolysis of the samples. Blood gases were also acquired over the period of treatment in the department. Several of the parameters included within the blood gas also could not be assessed due to hemolysis. This may have been due to coagulopathy related to hepatic injury and resultant dysfunction or difficulty during acquisition of the samples, similarly due to the instability of the patient. Of note, there was significantly elevated serum lactate on presentation which increased with time, from 16 o 20 mmol/L (reference range 0.5-2.2 mmol/L). Simultaneously, serum potassium rose from 5.4 to 6.6 mmol/L (reference range 3.5-5.3mmol/L) and hemoglobin levels fell from 73 to 56 g/L (reference range 130-180 g/L) indicating multi-organ dysfunction due to overwhelming infection.

**Fig. 1:**
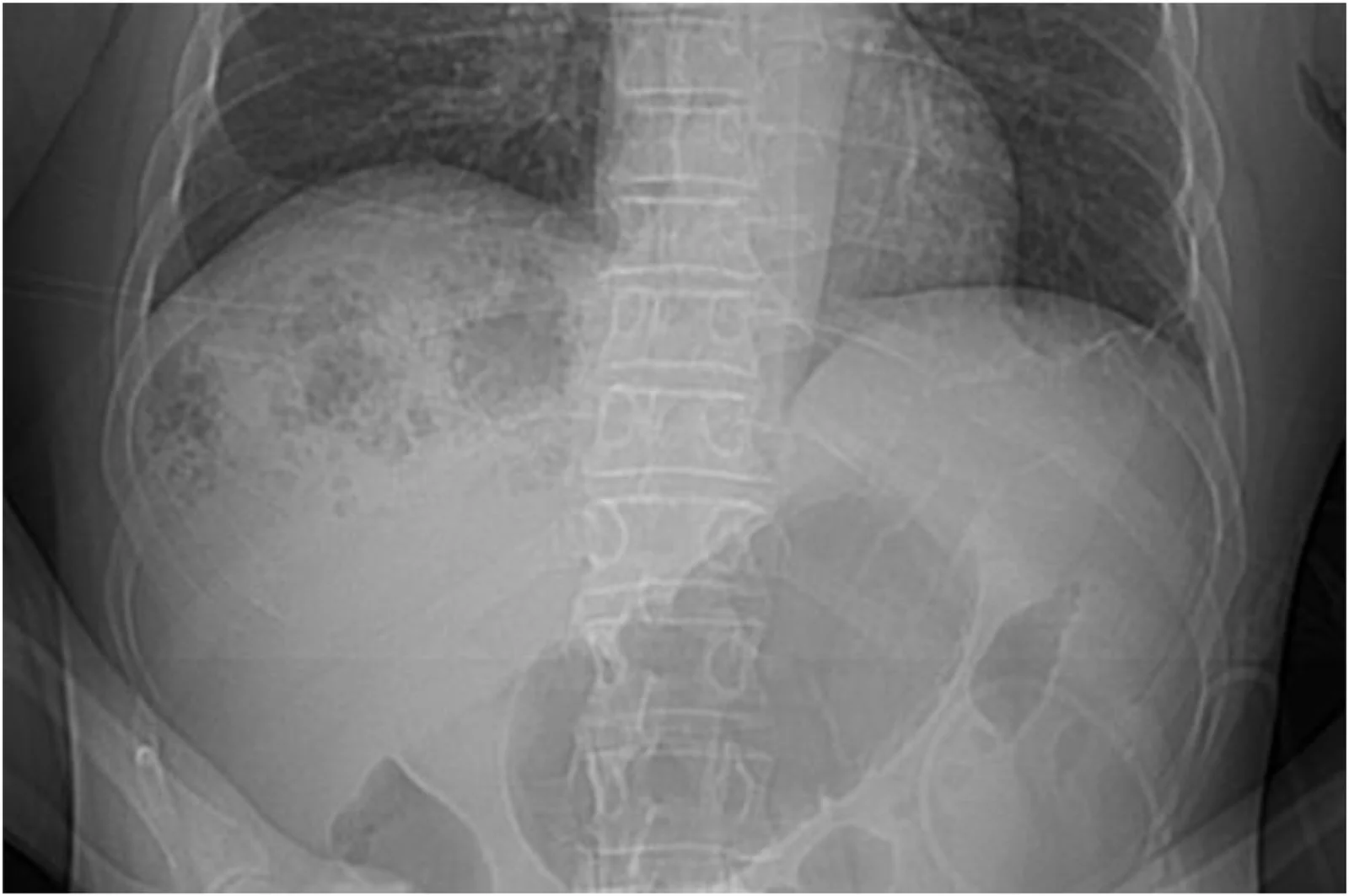
Coronal scout view from the CT scan. There are patchy areas of gas density in the right upper quadrant projected over the liver.

**Fig. 2:**
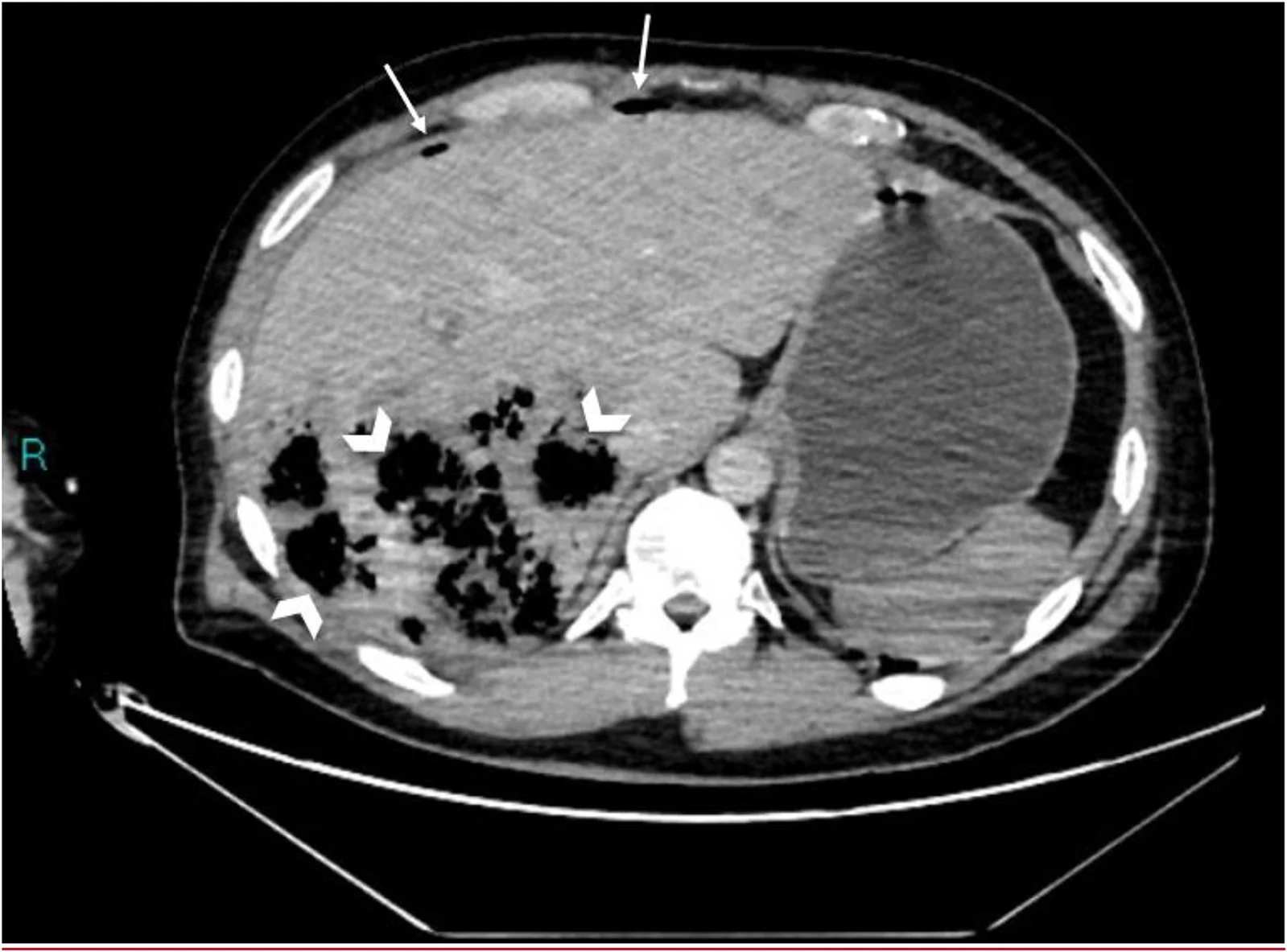
Axial CT section through the superior aspect of the liver. There is a small volume of pneumoperitoneum anterior to the liver indicating breach of the liver capsule (white arrows). Multiple gas-filled areas in segment VII and VIII of the liver (white arrow heads) without wall enhancement.

**Fig. 3:**
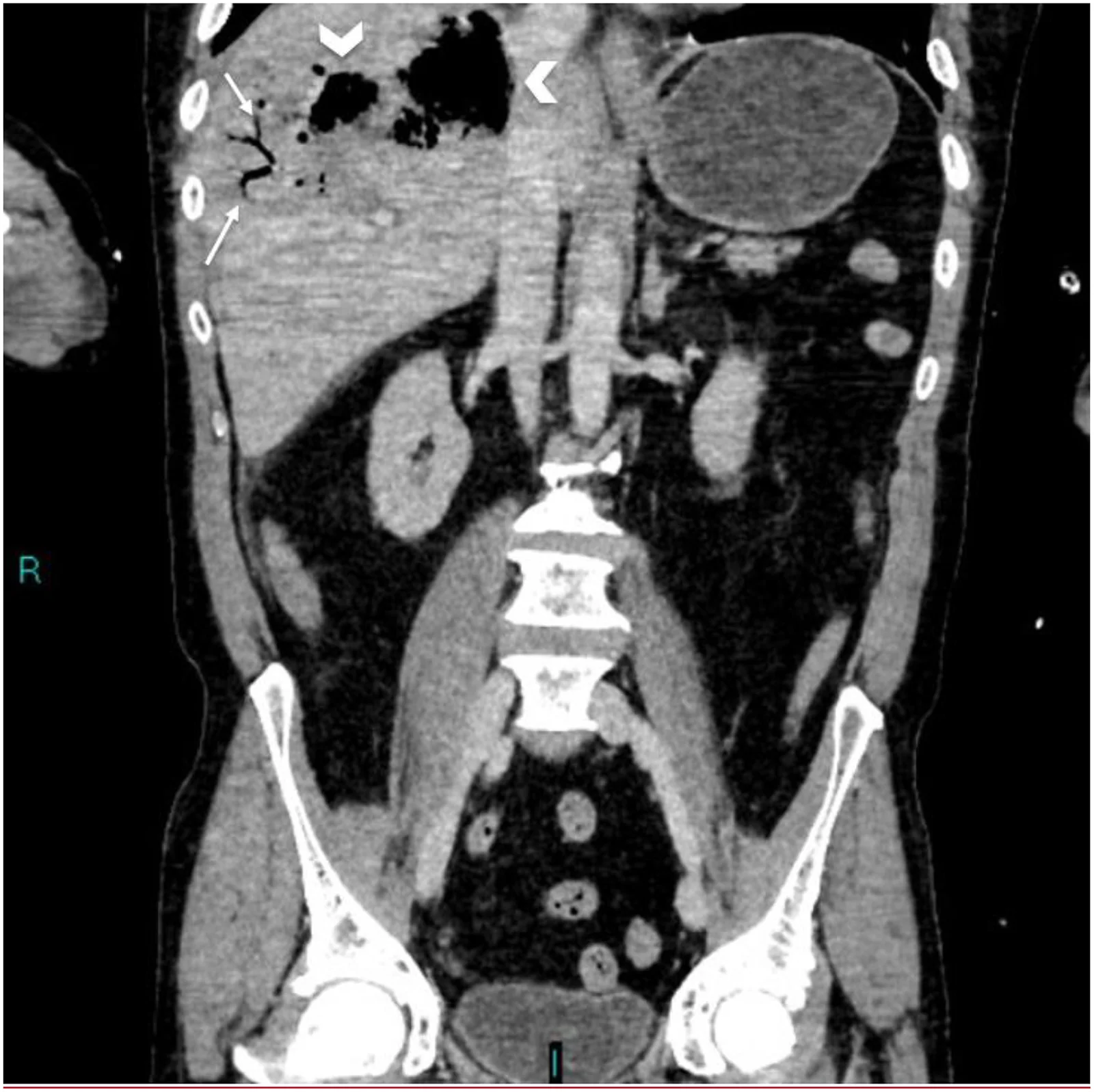
Coronal CT section through the posterior aspect of the liver. There are multiple gas-filled areas in segment VII and VIII of the liver (white arrow heads) without wall enhancement. Gas can be seen tracking along the portal vein branches (white arrows).

Within 45 minutes of presentation, the treating team had requested a CT examination of the abdomen and pelvis with portal venous phase contrast with a further 45 minutes passing before this was performed. The reporting Radiologist provided a verbal report to the clinical team 30 minutes following completion of the study with the final report made available to the team approximately 1 hour after the study was completed (Fig. 1, Fig. 2, Fig. 3).

The CT demonstrated imaging findings compatible with emphysematous hepatitis. Despite aggressive treatment with intravenous antibiotics and fluids, the patient continued to deteriorate. Initially metronidazole and gentamicin were administered with a subsequent dose of amoxicillin added. The clinical team had discussed the possibility of image-guided drainage as the next step in management. This would have required the patient to be transferred to an alternative Hospital as the presentation occurred overnight. The teams felt that at this time the patient was not stable enough to be transferred but should his condition improve then this remained the best next step in management. Surgical intervention under general anesthetic was considered but, due to severe hemodynamic instability and continued clinical deterioration, was not felt possible. Sadly, the patient’s condition deteriorated rapidly, and, despite several rounds of cardiopulmonary resuscitation, the patient succumbed to the aggressive infection. There was an attempt to resuscitate the patient following initial loss of cardiac output. Cardiopulmonary resuscitation was first performed approximately 90 minutes following the completion of the CT study, around 3 hours after presentation to the Emergency Department.

Blood cultures taken at the time of presentation ultimately grew 2 Gram positive organisms, commonly found in the gastrointestinal tract, *Enterococcus faecium* and *Clostridium perfringens.* There was no evidence in either the history, or from the imaging to suggest an alternative source for the infection which could have spread from a distant organ such as colitis or cholecystitis.

## Discussion

Our case demonstrates similarities with the previously described case reports of emphysematous hepatitis including the rapid progression of the disease, a relatively non-specific but typical presentation and the culture of bacteria consistent with others in the literature [5]. Whilst our patient had a history of T2DM, this was well controlled. Other cases report a spectrum of this with some patients not known to have T2DM and some with poorly controlled blood glucose levels [6].

Though it was not possible to compare or review many of the more commonly acquired biochemical markers, this suggests that severe cases of emphysematous hepatitis are likely to result in significant hepatic dysfunction as well as compromise of other organ systems, notably the kidneys in our case.

The CT images demonstrate typical appearances with replacement of the hepatic parenchyma with gas without a significant fluid component or enhancing wall. At the time of imaging, there was only segmental involvement of the liver with segments VII and VIII affected. The gas that had also invaded into the portal venous system was similarly confined to the same segments. There was no intrahepatic gas elsewhere with the infection localized to a concentrated area. There are several pathologies that commonly cause gas within the liver, the majority of which are indicators of significant underlying disease.

Pneumobilia—This would involve branching linear patterns of gas along the bile ducts. Typically, this has a central distribution with a preference for the left lobe of the liver. This is commonly seen following intervention, for instance post-ERCP or CBD stenting but can be due to other causes such as an incompetent sphincter of Oddi which again may be post-ERCP but also due to previous gallstone passage or pancreatitis. It would not be expected to cause a large cavity.

Portal venous gas—This is most seen in the context of ischemic bowel. Gas travels into the wall of the bowel due to translocation of bacteria from the lumen following the breakdown of the bowel wall as a result of ischemia. Gas can often be seen within the splanchnic veins as it travels to the liver via the portal vein. The pattern of gas distribution is also linear and branching but the distribution is typically peripheral as it follows the direction of the portal venous blood. This would usually be accompanied by evidence of bowel ischemia such as hypo or nonenhancement of the bowel wall and bowel dilatation. The underlying cause of the ischemia may also be evident such as thrombus in a splanchnic artery or a closed loop small bowel obstruction.

Postablation—During the treatment of liver lesions such as colorectal liver metastases, a high energy current is applied to the site of concern causing tissue necrosis. A pocket of gas is commonly seen at the site of the ablation immediately and persists for a few weeks before slowly resolving. Occasionally during treatment, gas can also be found in the portal veins and bile ducts, but this is a transient finding.

Hepatic infarction—Though rare due to the dual blood supply to the liver, hepatic infarction can occur and can lead to intraparenchymal gas. Most cases of hepatic infarction are due to acute occlusion of the portal vein as this supplies most of the blood to the liver. This usually happens in the context of hepatic transplantation or as a consequence of other hepatobiliary surgery. Appearances are initially of a wedge-shaped peripheral region of hypoattenuation. Gas is created in 2 ways, one of which is by secondary infection of the necrotic tissue but also as the necrotic tissue breaks down and releases intracellular gas, a cavity can form.

Gas forming pyogenic liver abscess—This is the main differential in this case. Bacteria such as *Klebsiella pneumoniae, Escherichia coli*, and *C perfringens* are implicated though they usually arrive at the liver from another site such as the colon in diverticulitis or the gallbladder in cholecystitis. The cavity formed by the abscess will contain a fluid/gas level as the gas produced by the breakdown of sugars and acids rises above the pus. Abscess cavities often have an irregular contour with enhancement of the periphery.

In our case, there are both linear branching regions of gas attenuation and larger cavities filled with gas. The cavities are entirely gas-filled with no fluid-level. There is no enhancement either within or around the periphery of the lesion and on review of the remainder of the abdomen and pelvis, there was no evidence of an alternative source of inflammation or infection. There was no reported previous surgery or intervention of any sort in our patient with patent portal veins and hepatic arteries leading to emphysematous hepatitis as the logical diagnosis.

Of the 2 organisms identified in our patient, *E faecium* and *C perfringens*, only the latter is a common gas-producing organism. This process occurs through the production of carbon dioxide and hydrogen gas through breakdown of carbohydrates. *C perfringens* is a rapidly progressing and aggressive organism which creates gases by the breakdown of glucose though is not associated with acid production [7]. Exotoxins produced by the bacteria cause rapid cell wall breakdown and cellular necrosis, resulting in carbohydrate availability and an anaerobic environment [8]. *E faecium* also results in the breakdown of glucose into its constituents but results in the formation of acids, rather than gases [9].

The aggressive nature of the infection is highlighted by the presence of pneumoperitoneum indicating that the liver capsule has been breached. This has been documented in 2 other cases with both proving fatal suggesting that the presence of pneumoperitoneum is a poor prognostic indicator, felt to represent advanced disease [10]. The presence of pneumoperitoneum is likely to confer a poor prognosis both due to the local extent of the liver destruction resulting in increased intraparenchymal pressures as well as the consequence of infective contents distributing throughout the peritoneal cavity and leading to overall more widespread pathology.

There have been a handful of documented cases of pneumoperitoneum in the context of gas forming hepatic abscess. This occurs through progressive enlargement of the abscess cavity which contacts the liver capsule before reaching a sufficient size and generating sufficient pressure that there is frank rupture of the capsule and release of gas into the peritoneal cavity [11]. This is somewhat analogous to the mechanism of pneumoperitoneum in pneumatosis cystoides intestinalis for instance whereby increasing pressure within gas filled cysts eventually reaches a threshold that results in rupture of the bowel wall [12]. This contrasts with pneumoperitoneum in the context of bowel perforation. This is commonly caused by a small hole being created across the entirety of the bowel wall for instance in cases of post-endoscopy pneumoperitoneum, peptic ulcer disease and diverticulitis following the creation of an initial microperforation due to trauma, inflammation or infection [13].

There are 4 other cases of pneumoperitoneum associated with emphysematous hepatitis in the published literature [4,10,14,15]. Aside from the extensive gas formation within the liver, there is not a consistent pathogen to link these cases though 2 do involve *Clostridia* spp*.*, one of which was *C perfringens.* Interestingly, in 2 of the 4 cases, the patient’s survived to hospital discharge despite the finding of pneumoperitoneum.

Treatment is usually a combination of intravenous antibiotics followed by early image-guided drainage with some cases then requiring or attempting surgical laparotomy. A single case was managed with intravenous antibiotics alone, though the area of gas within the liver was considerably smaller than in other reported cases highlighting the importance of early detection and intervention [4].

There remains a paucity of information in the published literature about this rare and fulminant entity. It is likely that overall numbers of cases are under-reported due to the rapid progression of the infection resulting in patient mortality before a diagnosis can be made. Cases in literature may also be lower due to high mortality of the condition, leading to increased difficulty in acquiring consent for publication. However, the number of reported cases in the literature does appear to be increasing. We initially performed a PubMed search using the keyword “Emphysematous Hepatitis” to include all published articles prior to February 2026. To our knowledge, there is no alternative or synonymous name for this entity. Articles were discounted if they did not discuss emphysematous hepatitis as the primary pathology, for instance some cases of pyogenic liver abscess were noted. Only cases in humans were considered, cases in other animals were excluded. Several articles made reference to further publications which were independently reviewed and assessed. This yielded 25 cases of emphysematous hepatitis. Of the now 26 cases reported, there have been 17 published since 2020 with only 9 reported in the previous 18 years following the first report in 2002. Potential reasons for this are likely to include an increase in the prevalence of T2DM, the most common association, as well as quicker and more widespread access to CT imaging which is a key tool in making a reliable diagnosis. In the UK alone, figures release for 2020/21, indicated that there were 2.65 million emergency CT scans performed whereas in 2024/25 this number has increased to approximately 4 million [16,17]. Not only is there a greater volume of emergency CT imaging being performed but also this is being performed earlier on in the patient journey allowing the diagnosis of rapidly progressing disease or deteriorating patients to be made. These factors allowing specialties such as Interventional Radiology and General Surgeons to make earlier and more focused management decisions with a greater level of detail for the exact nature of the underlying pathology, especially in cases with unusual presentations or rare pathologies. It is helpful to them to determine the most appropriate intervention, whether that be conservative, less invasive in the context of percutaneous drainage, or surgical intervention.

## Patient consent

The authors declare that they have discussed the case with the patient’s next of kin who has reviewed the manuscript and has given consent for publication (the patient is deceased).
